# Infections in Disorders of Immune Regulation

**DOI:** 10.3390/pathogens13030259

**Published:** 2024-03-17

**Authors:** Abarna Thangaraj, Reva Tyagi, Deepti Suri, Sudhir Gupta

**Affiliations:** 1 Pediatric Allergy Immunology Unit, Advanced Pediatrics Centre, Postgraduate Institute of Medical Education and Research, Chandigarh 160012, India; dr.abarna.t@gmail.com (A.T.); reva07tyagi@gmail.com (R.T.); 2 Division of Basic and Clinical Immunology, Department of Medicine, University of California, Irvine, CA 92697, USA; sgupta@hs.uci.edu

**Keywords:** ALPS, autoimmunity, STAT3 GOF, opportunistic infection, immune dysregulation, lymphoproliferation, Treg

## Abstract

Primary immune regulatory disorders (PIRDs) constitute a spectrum of inborn errors of immunity (IEIs) that are primarily characterized by autoimmunity, lymphoproliferation, atopy, and malignancy. In PIRDs, infections are infrequent compared to other IEIs. While susceptibility to infection primarily stems from antibody deficiency, it is sometimes associated with additional innate immune and T or NK cell defects. The use of immunotherapy and chemotherapy further complicates the immune landscape, increasing the risk of diverse infections. Recurrent sinopulmonary infections, particularly bacterial infections such as those associated with staphylococcal and streptococcal organisms, are the most reported infectious manifestations. Predisposition to viral infections, especially Epstein–Barr virus (EBV)-inducing lymphoproliferation and malignancy, is also seen. Notably, mycobacterial and invasive fungal infections are rarely documented in these disorders. Knowledge about the spectrum of infections in these disorders would prevent diagnostic delays and prevent organ damage. This review delves into the infection profile specific to autoimmune lymphoproliferative syndrome (ALPS), Tregopathies, and syndromes with autoimmunity within the broader context of PIRD. Despite the critical importance of understanding the infectious aspects of these disorders, there remains a scarcity of comprehensive reports on this subject.

## 1. Introduction

Primary immune regulatory disorders (PIRDs), a subset of human inborn errors of immunity (IEIs), diverge from classical immune deficiencies by predominantly manifesting as lymphoproliferation, autoimmunity, and malignancies rather than recurring infections. The 2022 update of the International Union of Immunological Societies (IUIS) Phenotypical Classification identifies 485 IEI-causing genes, with 45 genes classified under PIRDs, further categorized into seven distinct groups [1,2].

Advances in next-generation sequencing technologies have expanded our understanding of monogenic defects associated with PIRDs. According to the European Society for Immunodeficiencies (ESID) registry, PIRDs represent approximately 5.3% of all IEIs [3]. Unlike classical immune deficiencies, PIRDs are characterized by the loss of normal inflammatory control and self-tolerance mechanisms, leading to autoimmune manifestations such as cytopenias, enteropathy, lymphoproliferation, and malignancies. Although infections are less common, they can complicate the course of the disease or arise as a consequence of immunosuppressive therapies used in the management of immune dysregulation. Analyzing data on a total of 16,486 patients from the European Society for Immunodeficiencies registry, of which 1018 (19%) patients had an immune dysregulatory disorder, revealed that the most frequent initial manifestation was immune dysregulation without infection, occurring in 47% of patients. However, infection without dysregulation was prominent in 25% of patients, and both coexisted in another 14% [4].

While recent reviews have extensively covered the pathogenic mechanisms, clinical profiles, and management of common PIRDs [5,6], this review will specifically delve into the intricate relationship between infections and syndromes with autoimmunity. Including all immune dysregulatory disorders is beyond the scope of the review, so we have focused only on syndromes with autoimmunity with and without T regulatory defects. Increasing awareness among specialists and advocating for a multidisciplinary team approach, we aim to underscore the potential for early diagnosis of infections, thereby mitigating end-organ damage associated with these complex disorders.

### 1.1. Autoimmune Lymphoproliferative Syndrome (ALPS)

Autoimmune lymphoproliferative syndrome (ALPS), characterized by chronic lymphoproliferation, autoimmune manifestations, and an increased lymphoma risk, stems from defects in lymphocyte apoptosis, particularly in components of the apoptotic pathway (e.g., FAS, FASL, FADD, CASP10, and CASP8). While germline or somatic mutations in the FAS gene account for a majority of ALPS cases, approximately 20% remain genetically unidentified. The hallmark features include the presence of CD3+TCR ß+CD4-CD8- double-negative T cells (DNTs) and elevated plasma biomarkers such as interleukin-10 (IL-10), soluble Fas ligand (sFasL), and vitamin B12.

Despite a generally preserved cellular response to infection, ALPS patients have decreased circulating memory B cells and many have reduced responses to polysaccharide antigens [7]. Notably, splenectomy and neutropenia in ALPS patients elevate the risk of infections, with *Streptococcus pneumoniae* being a common pathogen. The decrease in neutrophils, low IgM levels, and diminished memory B cell counts in the spleen contribute to increased susceptibility, particularly to streptococcal infections [7,8].

Several cohorts, including a French cohort in 2011 and a study by Price et al. in 2014 from the National Institutes of Health (NIH), highlight the association between splenectomy and severe bacterial infections in ALPS [8,9], as summarized in Table 1. Factors such as age at the time of splenectomy and adherence to antimicrobial and vaccine prophylaxis play a significant role in infection causation, as evidenced by increased mortality rates and episodes of sepsis.

### 1.2. Fas-Associated Death Domain (FADD) Deficiency

Fas-associated death domain (FADD) deficiency is part of the programmed cell death complex; however, these patients have an increased predisposition to viral encephalitis rather than lymphoproliferation and autoimmunity [10]. The viral infections reported include varicella HHV6, EBV, CMV, HSV, astrovirus, and parainfluenza and can also occur after a live viral vaccine (such as MMR) [10,11,12,13]. There are reports of bacterial infections due to pneumococcal sepsis caused by functional hyposplenism. The patients who had lymphoproliferation were noticed to have increased numbers of DNT cells and various autoantibodies. These patients had significant mortality before 5 years of life [10,11,12]. 

### 1.3. Caspase-8 Deficiency State (CEDS)

Caspase-8 deficiency state (CEDS) is a relatively recently identified IEI. Caspase-8, encoded by the *CASP8* gene, initiates the extrinsic pathway of apoptosis. Caspase-8 is downstream of cell surface death receptors—FAS. Following activation of FAS, procaspase-8 is recruited by the death-effector domain (DED) heterodimer, and caspase-8 is ultimately activated [14]. Thus, deficiency of caspase-8 leads to a range of clinical manifestations like ALPS [14,15,16]. Caspase-8 is essential for T cell activation (specifically TCR-induced NF-kB activation), in addition to death receptor-mediated apoptosis. Caspase-8 deficiency affects the translocation and translation of NF-κB and further lymphocyte activation [14]. Caspase-8 also regulates necroptosis and pyroptosis, which are inflammatory lytic cell death pathways [17]. Therefore, apart from lymphoproliferation, CEDS is associated with profound immunodeficiency and manifests as recurrent infections, inflammation, and autoimmunity [14,15,16,18]. 

Initially described in siblings exhibiting lymphoproliferation and splenomegaly, CEDS has since been associated with a broader array of clinical features [19]. Very-early-onset inflammatory bowel disease with fistulating perineal disease presenting with chronic diarrhea and mimicking cow milk protein has been described. Other autoimmune manifestations like hemolytic anemia and interstitial lung disease have also been described [14,15,16,18,19]. Recurrent mucocutaneous herpetic infection was reported as a cardinal infection in patients with CEDS [19]. Recurrent sinopulmonary bacterial infections and viral infections like herpes, EBV, and molluscum contagiosum are also described [15,16,18,19,20] (Table 2). Fatal Nocardia meningoencephalitis has also been reported in a patient [16]. Serum immunoglobulin levels could be in the normal range or low, but the specific antibody response is insufficient. Despite normal T cell counts, pediatric patients had reverse CD4+ /CD8+ ratios [16,18,19]. These patients are managed with IVIg replacement therapy and acyclovir prophylaxis [16,19]. One patient was transplanted and seemed well on follow-up [18].

## 2. Syndromes with Autoimmunity Due to Treg Defects

Regulatory T cells (Tregs) play a major role in immune homeostasis by preventing or limiting T cell activation, particularly in the context of autoantigens [21,22,23]. Expression of the transcription factor forkhead box P3 (FOXP3), considered a master regulator of Treg development and function, is essential for their role in the maintenance of dominant tolerance [24]. Regulatory T cells develop primarily in the thymus, although they can also be differentiated in the periphery. The delineation of these two populations in the peripheral Treg compartment is difficult due to the lack of specific markers. The development of thymus-derived Tregs is known to require high-avidity interaction with MHC-self peptides, and they are major contributors to self-tolerance [21,22,23]. FOXP3 expression is stimulated by IL-2 and IL-15 cytokines [24]. Peripheral Treg development is less clearly understood and may be influenced by factors such as stimulation from intestinal commensal microbiota, chronic exposure to antigens in small dosages, and various environmental stimuli. Peripheral Tregs contribute to the suppression of immune responses to common nonpathogenic stimuli.

Treg cells function by suppressing effector T cell activities, thereby controlling uncontrolled proliferation, proinflammatory cytokine production, growth factor expression, and costimulatory molecule expression. Disorders affecting the number, function, and stability of Tregs are termed “Tregopathies”. These disorders can be classified into three categories: Treg developmental defects due to FOXP3 deficiency, as seen in immune dysregulation, polyendocrinopathy, enteropathy, X-linked syndrome (IPEX) and BACH2 deficiency; Treg stability defects related to impaired IL-2 signaling (involving CD25 and CD122); and Treg functional defects (such as CTLA-4 HI, LRBA, DEF6, and STAT3 GOF). While other genes are implicated in Treg defects, such as STAT5b, STAT1 LOF, ITCH, ITK, RAG1, and RAG2 deficiency, they are not discussed here, as they are not included in the IUIS 2022 classification of syndromes with autoimmunity due to Treg defects [1,2].

### 2.1. Treg Developmental Defects

#### 2.1.1. Immune Dysregulation, Polyendocrinopathy, Enteropathy, X-Linked (IPEX) Syndrome

Immune dysregulation, polyendocrinopathy, enteropathy, X-linked (IPEX) syndrome is characterized by enteropathy, severe dermatitis, and autoimmune endocrinopathies due to hemizygous mutation in the FOXP3 gene. FOXP3 is a transcriptional factor that is primarily expressed by CD4+CD25+ Tregs and controls their function and maintenance.

The majority of patients present between 1 month and 1 year, with a few having delayed onset of more than 1 year of age [25]. A comprehensive study of 96 patients with IPEX showed a classical triad of type 1 diabetes, eczema, and enteropathy as the most common manifestations. Apart from the classical triad, other manifestations include failure to thrive, alopecia, dermatitis, arthritis, autoimmune pancreatic exocrine insufficiency, gastritis, kidney disease, interstitial lung disease, and infection [25,26]. Table 3 summarizes the studies reporting infections in patients with IPEX syndrome. These patients also predominantly develop staphylococcal sinopulmonary infections as well as candidal infections. Rapamycin, which is an mTOR inhibitor, is a targeted therapy for the management of immunological complications in IPEX. It helps in the restoration of Treg cell function in IPEX patients [27].

#### 2.1.2. BACH2 Deficiency

Autosomal dominant immunoregulatory disorder due to BACH2 deficiency results in decreased protein expression in B and T cells. BACH2 is involved in the downstream signaling of T cell receptors, regulation of Th2 cytokine production, and stabilization of Treg cells. Haploinsufficiency leads to recurrent sinopulmonary infections, lymphoproliferation, and enteropathy due to decreased total and switched memory B cells, hypogammaglobulinemia, and reduced Treg cells [29]. Afazali et al. described a father–daughter dyad presenting with a CVID-like phenotype with sinopulmonary infections secondary to severe hypogammaglobulinemia [29].

### 2.2. Treg Stability Defects

#### 2.2.1. Deficiency of CD25

Autosomal recessive deficiency of IL-2 receptor alpha chain or CD25 leads to an IPEX-like syndrome. IL-2 is required for both the initiation and maintenance of adaptive T cell responses and the survival and function of FOXP31 Treg cells. Manifestations include enteropathy, autoimmune manifestations, and lymphoproliferation [30,31,32]. Impaired Treg and T cell function underlies the clinical picture. While sinopulmonary, skin, and soft tissue infections due to *Staphylococcus aureus* are most common, fungal infections with candida and Aspergillus pneumonia as well as viral infections with CMV and varicella have also been described. Vignoli et al. described a 2-month-old boy with gastrointestinal infection with Pseudomonas and human herpesvirus 6 [33].

#### 2.2.2. CD 122 Deficiency

Interleukin 2 (IL2) acts via its receptor, which has alpha, beta, and gamma subunits. Interleukin-2 receptor (IL2R) gamma deficiency leads to T–B+NK- SCID, while IL2R beta (CD122) deficiency is associated with an IPEX-like phenotype [34]. It is characterized by increased memory T cells and NK cells, decreased CD4+CD25+FOXP3+ Tregs, and hypergammaglobulinemia. Severe dermatitis, thyroiditis, enteropathy, and lymphocytic interstitial pneumonia are observed, along with serious CMV and EBV infections [34,35].

### 2.3. Treg Functional Defects

#### 2.3.1. Lipopolysaccharide-Responsive Beige-like Anchor Protein (LRBA) and Cytotoxic T Lymphocyte Antigen-4 (CTLA-4) Defects

The deficiency of CTLA-4 is known as CTLA-4 haploinsufficiency with autoimmune infiltration (CHAI), while LRBA defects are termed “LRBA deficiency with autoantibodies, regulatory T (Treg) cell defects, autoimmune infiltration, and enteropathy” (LATAIE), emphasizing immune dysregulation as a major feature [36]. Both LRBA and CTLA-4 were identified as causes of monogenic common variable immunodeficiency (CVID) in 2012 and 2014, respectively [37].

CTLA-4 serves as a crucial checkpoint in T cell functions, expressed both as a cell surface molecule and in a soluble form in all T cells; however, it is highly expressed in FoxP3+ Tregs. During T cell interaction with antigen-presenting cells (APC), CTLA-4 binds with CD80/CD86, competing with CD28 and leading to the downregulation of T cell receptors, subsequently decreasing IL-2 production and, thus, affecting effector T cell function. Haploinsufficiency of CTLA-4 results in the absence of T regulatory cell function, leading to autoimmunity. CTLA-4 is regulated by LRBA. LRBA regulates CTLA-4 by preventing its lysosomal destruction. So, deficiency of LRBA presents like CLTA-4 deficiency [38].

Mutations in CTLA-4 and LRBA result in low B cell numbers, a significant decrease in switched memory B cells, and hypogammaglobulinemia, leading to a CVID phenotype [39]. Additionally, there is a noticeable reduction in NK cells and CD8 T cells [40]. They also have a phenotypic presentation of severe hypogammaglobulinemia or an isolated decrease in Ig G or Ig A levels [41].

Autoimmune manifestations are the predominant features in CHAI and LATAIE. Jamee et al. reported 222 patients with CTLA-4 haploinsufficiency and 212 patients with LRBA deficiency [37]. Nearly 66.8% had at least one autoimmunity, and 47.7% were reported to have two or more autoimmune disorders. Autoimmune cytopenias were the most common autoimmune complications in CHAI and LATAIE patients in 67% and 70%, respectively. It includes autoimmune hemolytic anemia, Evans syndrome, idiopathic thrombocytopenic purpura, and autoimmune neutropenia [36,42]. Even though chronic diarrhea or inflammatory bowel disease was seen in both, it is more common among LATAIE. The other autoimmune manifestations reported include alopecia, vitiligo, autoimmune thyroiditis, Addison’s disease, arthritis, hepatitis, vasculitis, etc. Lymphoproliferation is seen in around 40–50% of the patients [36].

#### 2.3.2. Cytotoxic T Lymphocyte Antigen-4 (CTLA-4) Defects

In CTLA-4, infection rates are slightly lower (50–60%) compared to LRBA defects [36]. Sinopulmonary infections, including those caused by *H. influenzae*, *Streptococcus pneumoniae*, and *Staphylococcus aureus*, are predominant, reflecting the impaired immune response against these common pathogens. Respiratory viruses also contribute to the infectious profile. Furthermore, rare instances of mycobacterial infections, particularly pulmonary and esophageal tuberculosis, have been documented in a subset of patients [43]. Herpes infections, including those induced by EBV, have emerged as characteristic features, often leading to lymphoproliferative disorders [43,44]. A retrospective study reported malignancies in CTLA-4 deficient patients, with EBV-induced Hodgkin lymphoma observed in a notable proportion [45]. Fungal infections such as candida and aspergillosis have been reported, underscoring the diverse infectious challenges faced by individuals with CTLA-4 deficiency [43]. The compromised immune regulatory functions of CTLA-4 contribute to the complex infectious landscape in affected individuals, necessitating comprehensive management strategies that address both the primary immunodeficiency and associated infections.

Intravenous immunoglobulin (IVIg) and antibiotic prophylaxis are used for the prevention of infection. However, the mainstay of treatment for CLTA-4 is immunosuppressive therapy with abatacept and corticosteroids [36,43,45]. Other drugs like rituximab, mycophenolate mofetil, cyclosporine, and azathioprine have also been used for the management of immunological manifestation. Splenectomy has been offered for patients with refractory cytopenia, but HSCT is used as curative therapy [36,43,45,46].

#### 2.3.3. Lipopolysaccharide-Responsive Beige-like Anchor Protein (LRBA)

Infections occur in approximately 50–80% of individuals with LRBA defects [47,48,49], mainly manifesting as recurrent sinopulmonary infections involving viral and bacterial agents [36,47,49]. Other reported infections include aspergillus pneumonia, salmonellosis, candida, amoebiasis, gastrointestinal infections (e.g., giardia, H. pylori, Trichomonas hominis), and strongyloidiasis [50]. Viral infections involve respiratory viruses, human papillomavirus-causing warts [50], CMV, and EBV, which can lead to lymphoproliferative disorders and malignancies [48,51], including BK virus-induced nephropathy [51] (Table 4).

Immunosuppressive drugs are used for the management of LRBA. Complete remission in lymphoproliferation, immune dysregulation, chronic diarrhea, and cytopenia were noticed in LRBA patients with abatacept therapy [40]. Other drugs like azathioprine, tacrolimus, methotrexate, tumor necrosis factor (TNF) alpha inhibitors, and cyclosporine have been used; however, HSCT is the mainstay of treatment. IVIg and antibiotic prophylaxis are used for infectious complications [36]. 

#### 2.3.4. Deficiency of Differentially Expressed in FDCP6 Homolog (DEF6)

DEF6 gene mutation affects CTLA-4 regulation, resulting in functional CTLA-4 defects [28]. The seven reported cases exhibit recurrent infections, immune dysregulation, EBV susceptibility, and malignancy. Reduced naïve CD4T cells, reversed CD4/CD8 ratio, diminished vaccine responses, and hypogammaglobulinemia characterize DEF6 mutations. Immune dysregulation includes autoimmune hemolytic anemia, recurrent colitis, arthritis, synovitis, and positive ANCA, DCT, and APLA [39,53,54]. Infections range from Streptococcus, Staphylococcus, and Enterococcus spp. to viral infections like CMV, EBV, influenza, rhinovirus, RSV, and rotavirus. Severe fungal infections with Malassezia furfur have been reported [53,54]. A patient with EBV infection resulting in lymphoproliferation and Hodgkin lymphoma has also been described [53].

### 2.4. STAT3 Gain of Function Mutation

Activated by IL-6 and IL-10 family members, IL-21, IL-27, G-CSF, leptin, and IFN, STAT3 primarily regulates the immune response, cell growth, differentiation, apoptosis, and tumor occurrence [55]. In cases of STAT3 GOF, there is reduced production of Tregs, leading to autoimmunity. Clinical presentations often include failure to thrive, type 1 diabetes mellitus, inflammatory colitis, interstitial lung disease, and other autoimmune manifestations [56].

Infections occur in approximately 20–50% of STAT3 GOF patients, with recurrent respiratory tract infections being the most common [57]. A multicentric study by Leiding et al. identified a high infection rate of 72%, with bacterial infections (80%), viral infections (61%), and fungal infections (25%) being prevalent. Opportunistic and mycobacterial infections are rarely reported (7% and 6%, respectively) [58] (Table 5).

Milner et al. reported an increased risk of recurrent sinopulmonary infections, candidiasis, recurrent herpes infection, varicella, molluscum infection, disseminated Pseudomonas, and Serratia urinary tract infection in 13 cases with STAT3 GOF [59]. The spectrum of infections is hypothesized to be linked to nonspecific hypogammaglobulinemia, variable T, B, and NK cell lymphopenia, reduced functional responses to mitogens, reduced Tregs, and elevated double-negative T cells and IL17-producing T cells [57].

For managing inflammatory complications, JAK inhibitors and IL-6 inhibitors are employed, but there are reports of associated infections such as respiratory viral infections, herpes infections, cryptococcal infections, and Pneumocystis pneumonia [60]. The delicate balance between immunosuppression and infection risk requires careful consideration in the management of these complex syndromes.

**Table 5:** Infection profile reported in patients with STAT3 GOF.

S No	Author/Year	Number of Patients	Infection	Associated Immunological Abnormalities
1	Erdős M et al., 2021 [61].	1	Recurrent sinopulmonary infection	Hypogammaglobulinemia, CD4+ lymphopenia
2	Gonzalez-Mancera MS et al., 2020 [62].	1	Recurrent sinus infection; bronchitis; ear infection; *M. abscessus* respiratory infection	Hypogammaglobulinemia
3	Gutiérrez M et al., 2018 [63].	2	Urinary tract infection—1/2; respiratory infection—2/2 (RSV—1/2); recurrent oral candidiasis—1/2	Hypogammaglobulinemia
4	Thalhammer J et al., 2021 [64].	1	Hepatitis C	Reduced naïve CD45RA+ T cells and naïve B cells
5	Lin L et al., 2020 [65].	12	Respiratory tract infection—11/12; abscess—8/12; Staphylococcal infection—8/12; otitis media—5/12; chronic mucocutaneous candidiasis—5/12	Reduced NK cells, reduced memory B cells, elevated DNT
6	Tanita K et al., 2021 [66].	8	Recurrent sinopulmonary infection—3/8; varicella—1/8	Hypogammaglobulinemia, T, B, and NK cell lymphopenia, CD4/CD8 ratio reversal
7	Milner et al., 2015 [59].	8	Viral infections—3/8 (herpes zoster—2/8, human papillomavirus—1/8, molluscum—1/8); respiratory tract and lung infection—3/8; bacterial and fungal soft tissue infection—1/; osteomyelitis—1/8; sepsis—1/8 (*Pseudomonas* and *Serratia*); candidal infection—2/8; UTI—1/8	B cell and T lymphopenia, low memory B cell counts, hypogammaglobulinemia

### 2.5. IKAROS Gain of Function

Ikaros is a member of a family of zinc finger transcription factors and plays a vital role in early hematopoietic development and differentiation into the three major hematopoietic lineages [67]. Four different clinical presentations based on the type of Ikaros mutation have been described [67,68,69,70,71,72].

Haploinsufficiency (HI) of Ikaros results in a CVID-like phenotype with recurrent sinopulmonary infections, such as pneumonia, otitis media, sinusitis, bronchitis, sepsis, and meningitis, as the predominant manifestations. Viral infections like HSV and HPV have been reported [69]. Dominant-negative (DN) mutations lead to a more severe combined immune deficiency phenotype, presenting early in life with Pneumocystis jiroverci pneumonia, bacterial infections (e.g., *S. pneumoniae*, *Klebsiella* spp., *Pseudomonas* spp.), and viral infections (e.g., RSV, HSV, HPV, adenovirus, influenza) [70,71]. There are reports of candida infections, pulmonary aspergillosis, and mycobacterial infections due to the Mycobacterium avium complex and Mycobacterium avium intercellulare. Viral infections like RSV, HSV, adenovirus, and influenza have been reported [70,71].

Dimerization defects (DDs) are associated with benign or malignant hematological conditions, autoimmune manifestations, and malignancies like T-ALL, B-ALL, and Burkitt lymphoma [73]. Low B cell counts and hypogammaglobulinemia contribute to infections in HI and DD, while DN mutations exhibit both reduced T and B cell counts and thus have a propensity to develop opportunistic infections as well as severe viral infections. Neutropenia and eosinopenia can also occur later in life due to the involvement of myeloid cells [67,69,70,73].

An IKAROS GOF mutation has been recently described in eight patients from four unrelated families. Autoimmune manifestations are seen in 75% of the population. These include type 1 diabetes mellitus, enteritis, autoimmune hepatitis, Hashimoto’s thyroiditis, leukocytoclastic vasculitis, vitiligo, alopecia, and cytopenia. It is also associated with recurrent sinopulmonary infection and otitis media [72].

### 2.6. FERMT1 Deficiency

The FERMT1 gene encodes proteins involved in integrin signaling and linkage of the actin cytoskeleton to the extracellular matrix. FERMT1 deficiency is characterized by recurrent blistering genodermatosis with photosensitivity and progressive poikiloderma known as Kindler syndrome [74]. Associated mucosal inflammation results in dental caries and gingivostomatitis and makes patients prone to oral candidiasis [75]. They also exhibit an increased risk for the development of nonmelanoma skin and oral cancers with considerable phenotypic variability. The precise mechanisms by which FERMT1 deficiency predisposes individuals to these infections may involve compromised immune responses, altered epithelial barrier function, or other immune system abnormalities [74,75].

## 3. Syndromes with Autoimmunity with or with Lymphoproliferation

### 3.1. Autoimmune Polyendocrinopathy-Candidiasis-Ectodermal Dystrophy (APECED)

Medullary thymic epithelial cells play a crucial role in the establishment of self-tolerance by expressing the AIRE gene, which acts as a critical checkpoint in immune tolerance development. Deficiency in the AIRE gene leads to autoimmune polyendocrinopathy-candidiasis-ectodermal dystrophy (APECED) syndrome. This syndrome is characterized by a classic triad of chronic mucocutaneous candidiasis (CMCC), hypoparathyroidism, and Addison’s disease.

CMCC, a hallmark feature of APECED, arises from autoantibodies targeting interleukin-22 (IL-22) and interleukin-17F (IL-17F). It is prevalent in approximately 50–90% of APECED patients and tends to increase with age [76]. A Russian study by Orlova et al. reported CMCC in nearly 75% of cases [77]. Beyond the triad, patients with APECED may also present with hypothyroidism, autoimmune hepatitis, pernicious anemia, autoimmune cytopenia, vitiligo, alopecia, and autoimmune involvement of other organs [76,77].

### 3.2. SOCS1 Deficiency

SOCS1 (suppressor of cytokine signaling 1) serves as a crucial negative regulator involved in various pathways of the immune system. It functions as an immune checkpoint within the JAK/STAT pathway, participates in ubiquitin-mediated proteasomal degradation, and influences the TCR signaling pathway [78]. Haploinsufficiency of SOCS1 leads to a diverse spectrum of autoimmunity characterized by multiple autoantibody positivity, with autoimmune cytopenia being the most common manifestation. Additionally, SOCS1 deficiency is associated with lymphoproliferation, malignancy, and heightened susceptibility to infections [78,79,80,81,82]. Thaventhiran et al. and Lee et al. have documented sinopulmonary infections and abscess formation attributed to *Streptococcus pneumoniae* and *Moraxella catarrhalis* in individuals with SOCS1 haploinsufficiency [80,83]. Furthermore, there are reports of varicella-zoster infections, shingles caused by herpes simplex virus, and susceptibility to COVID-19 infection observed in affected individuals [79,80,83].

### 3.3. ITCH Deficiency

ITCH (itchy E3 ubiquitin protein ligase) plays a critical role in proteasomal degradation by functioning as an E3 ubiquitin protein ligase. Its activity leads to the suppression of T cell-mediated immune responses. Initially described in 10 Amish children, ITCH deficiency manifests with a spectrum of symptoms including failure to thrive, dysmorphic features, developmental delay, lung disease, autoinflammation, and recurrent infections [84]. Developmental delay and autoimmunity are significant aspects of this disease [84,85,86]. Patel et al. reported the case of a 3-year-old boy with very-early-onset inflammatory bowel disease (VEO-IBD), severe arthritis, and uveitis associated with ITCH deficiency. The patient also experienced recurrent episodes of sinopulmonary infections and cutaneous abscesses. He was successfully treated by HSCT [86].

### 3.4. Prolidase Deficiency

Prolidase deficiency (PD) is a rare autosomal recessive disorder caused by mutations in the PEPD gene, which plays a crucial role in collagen breakdown, wound healing, and angiogenesis. The condition is characterized by a distinctive array of clinical features, including severe, chronic, and painful skin ulcers primarily affecting the lower extremities, as well as telangiectasias of the face and hands. Patients often experience recurrent infections, particularly involving the skin and respiratory tract, alongside dysmorphic facial characteristics, variable degrees of intellectual disability, and organomegaly. Further manifestations of PD may encompass skeletal abnormalities, chronic pulmonary disease, anemia, thrombocytopenia, elevated liver enzyme, hypergammaglobulinemia, hypocomplementemia, and autoimmune phenomena such as systemic lupus erythematosus (SLE) [87]. PD patients typically exhibit deficient PEPD activity, leading to impaired type I interferon receptor-dependent immune responses crucial for bolstering innate immunity and triggering adaptive immune defenses against infections [88].

Infections commonly observed in PD include bacteremia, skin infections, and cellulitis, with notable occurrences of influenza, *Pseudomonas aeruginosa*, and fungal infections, which are characteristic of combined or innate immune deficiencies [88,89,90,91]. Laboratory findings often reveal elevated levels of immunoglobulins (IgG, IgA, IgM, and IgE), complement component C1q deficiency, and reduced levels of C3 and C4 complement components. Hypergammaglobulinemia may arise from recurrent infections or immune dysregulation [88,89,91,92]. The association between PD and SLE is marked by shared clinical manifestations such as anemia, thrombocytopenia, hypergammaglobulinemia, hypocomplementemia, and increased autoantibody titers. Another potential mechanism linking PD to immune dysregulation involves the nuclear factor kappa B (NFκB) transcription factor, with prolidase activity inversely correlated with NFκB activity [88].

## 4. Activated Phosphoinositide 3-Kinase δ Syndrome (APDS)

Activated phosphoinositide 3-kinase δ syndrome (APDS) comprises two types: type 1 results from heterozygous gain-of-function mutations in the catalytic subunit of p110δ (PIK3CD), while type 2 arises from mutations in the regulatory subunit p85α (PIK3R1) [93,94]. These mutations lead to increased activity of the phosphoinositide-3-kinase δ (PI3Kδ) pathway. APDS manifests as a heterogeneous group of disorders, encompassing presentations such as Hyper IgM syndrome, common variable immunodeficiency (CVID), combined immunodeficiency (CID), autoimmune lymphoproliferative syndrome (ALPS), antibody deficiency, malignancy, or recurrent infections [95]. Recurrence of respiratory infections is the most common manifestation in nearly 65% of individuals with APDS, with lymphoproliferation observed in approximately 15% [95]. Notably, in APDS type 1, recurrent respiratory tract infections occur in nearly 98% of patients, followed by lymphoproliferation in 75%. Conversely, APDS type 2 is associated with upper respiratory tract infections in 100% of cases and lower respiratory tract infections in 77% [96,97,98]. Pathogens commonly implicated in APDS include *Streptococcus pneumoniae*, *Haemophilus influenzae*, *Staphylococcus aureus*, *Moraxella catarrhalis*, *Pseudomonas aeruginosa*, and the *Klebsiella* species, as well as viral infections such as Epstein–Barr virus (EBV), cytomegalovirus (CMV), herpes simplex virus (HSV), and varicella-zoster virus (VZV) [96,97,98]. EBV-associated lymphoproliferation has also been described with this disorder. There is no significant difference in the clinical and infectious spectrum between the two types; however, chronic diarrhea is reported more commonly with type 2 APDS, while APDS 1 is associated with other autoimmunity conditions [98].

Immunological abnormalities in APDS include low CD19+ B cell counts (74.8%), reduced T cell and natural killer (NK) cell counts (28.4% and 18.1%, respectively), and decreased CD4+CD45RA+ naive T cell counts in 88.3% of patients. Additionally, there is an increase in senescent T cell counts, reduced levels of IgG and IgA, and elevated IgM levels [95,96,97,99]. Treatment options for APDS include intravenous immunoglobulin (IVIg) replacement therapy and immunosuppressive agents such as steroids, rituximab, and sirolimus. Leniolisib, a PI3Kδ inhibitor, represents a novel therapeutic approach [96]. However, hematopoietic stem cell transplantation is the curative therapy.

## 5. Treatment of PIRD

Although management of PIRD predominantly focuses on addressing autoimmune manifestations, during acute infections, a combination of antibiotics and antiviral drugs is used. Like other IEIs, prevention of infections based on the clinical presentation and type and level of the immune deficiency is advocated. Chemoprophylaxis, specifically with trimethoprim/sulfamethoxazole or azithromycin, is used, especially in patients with recurrent sinopulmonary infections. Long-term antibiotic prophylaxis against pneumococcal sepsis using penicillin V or fluoroquinolones, such as levofloxacin, has been used in asplenic ALPS patients. In addition, education regarding the importance of seeking medical care promptly for a significant febrile illness requiring intravenous antibiotics until bacterial sepsis is ruled out has been emphasized in patients with ALPS who have undergone a splenectomy. Periodic surveillance and reimmunization against pneumococci using a combination of both conjugate (Prevnar-13, Prevnar-20) and 23-valent polysaccharide (Pneumovax) vaccines every 4 to 5 years have also been advised.

Immunoglobulin replacement therapy has been recommended for patients with hypogammaglobulinemia (LRBA deficiency and haploinsufficiency CTLA 4), while hematopoietic stem cell transplants (HSCTs) have been curative for patients with immune dysregulatory disorders [36,44,100,101]. Table 6 summarizes the use of immunomodulators, antibiotic prophylaxis, replacement immunoglobulins, and HSCT in these disorders.

To conclude, infections are infrequent compared to other IEIs, yet they are important presenting manifestations of these disorders. Table 7 summarizes the infection profile in immunedysregulatory disorders. They may manifest as part of the disease or as a consequence of therapeutic interventions targeting lymphoproliferation. Knowledge about the spectrum of infections in these disorders is critical for early diagnosis and optimum management.

## Figures and Tables

**Table 1:** Infection profile reported in large cohorts in patients with ALPS.

S No	Author/Year	Number of Patients	Infection	Associated Immunological Abnormalities
1	Neven et al., 2011 and 2014 [4,5]	12	Before splenectomy: invasive *S. pneumoniae*—2/12 After splenectomy: sepsis—10/12 (*S. pneumoniae*—4/12; *S. agalactiae*—1/12)	Hypogammaglobulinemia, low memory B cell and low marginal zone B cell counts
2	Price et al., 2014 [9]	150	After splenectomy: sepsis—27/66 (*S. pneumoniae*—19/27, *Haemophilus influenza*—2/27, *Capnocytophaga cynodegmi*—1/27); meningitis—6/66 (*S. pneumoniae* or *N. menigitidis*)	Post splenectomy

**Table 2:** Infections reported in patients with CEDS.

S No	Author/Year	Number of Patients	Infection	Associated Immunological Abnormalities
1	Chun HJ et al., 2002 [19]	2	Recurrent sinopulmonary infection Herpes simplex infection	IgM—low Specific antibody response poor CD4/CD8 ratio reversal
2	Niemela J et al., 2015 [16]	2	Pneumonia, Nocardia asteroids meningoencephalitis	IgM—low CD4/CD8 ratio reversal
3	Lehle AS et al., 2017 [20]	3	Sinopulmonary infection, conjunctivitis, skin and soft tissue infection, and molluscum contagiosum infection	Not available
4	Chougule A et al., 2023 [18]	1	*Klebsiella pneumoniae* in stool culture	IgM—normal Specific antibody response poor CD4/CD8 ratio reversal

**Table 3:** Infection profile reported in patients with IPEX syndrome.

S No	Author/Year	Number of Patients	Infection	Associated Immunological Abnormalities
1	Yu Huang et al., 2022 [26].	5	Recurrent respiratory tract infection—4/5 and oral thrush—1/5	Low levels of IgA, IgM CD4/CD8 ratio reversal
2	Gambineri et al., 2008 [28].	10	Severe ear infection—1/10	Elevated IgE levels

**Table 4:** Infection profile reported in large cohorts in patients with LRBA.

S No	Author/Year	Number of Patients	Infection	Associated Immunological Abnormalities
1	Kiykim et al., 2019 [40].	22	Recurrent sinopulmonary infection—19/22 Viral infection—13/22 Candidiasis—4/22 Giardiasis—2/22	Lymphopenia, neutropenia, hypogammaglobulinemia, reduced CD4+ and CD8+ T cells
2	Kostel Bal et al., 2017 [52].	7	Respiratory infection: coronavirus—3/7; adenovirus—2/7; RSV—2/7; *Staphylococcus aureus*—1/7 Sepsis: Acinetobacter—1/7; Enterococcus—1/7; Serratia marcescens—1/7; Candida—2/7 Invasive viral infection: CMV—2/7; EBV—1/7	Hypogammaglobulinemia
3	Cagdas et al., 2019 [51].	15	Upper respiratory tract infection—14/15 Lower respiratory tract infection—8/15 CMV infection—2/15 Recurrent herpes infection—1/15	Lymphopenia—both T and B cells, hypogammaglobulinemia.
4	Lopez-Herrera G et al., 2012 [48].	5	Respiratory infection—5/5 (*S. pneumoniae*) Abscess—1/5 (*Staphylococcus aureus* and *Streptococcus viridians*)	Hypogammaglobulinemia
5	Meshaal et al., 2020 [49].	18	Respiratory infection—5/18 Abscess—2/18 (brain abscess—1/8, skin abscess—1/8)	Hypogammaglobulinemia, T and B cell lymphopenia
6	Alkhairy et al., 2015 [50].	31	Respiratory infection—19/31 Infective diarrhoea—3/31(Giardia species, Trichostrongylus and Trichomonas hominis)	Hypogammaglobulinemia, low B cell counts

**Table 6:** Summary of use of chemoprophylaxis, immunoglobulin replacement therapy, and HSCT in patients with PIRD.

Diseases	Targeted Therapy	Chemoprophylaxis and Other Therapy	Immunoglobulin Replacement	Haematopoietic Stem Cell Transplant	Reference
ALPS	Sirolimus	Penicillin prophylaxis post splenectomy, Trimethoprim/sulfamethoxazole, Steroids, Mycophenolate mofetil, Cyclosporin A, Rituximab, Pentostatin, Sirolimus	+	+++	[7,8,9,102,103]
STAT3 GOF	JAK inhibitors, IL-6 inhibitors	Trimethoprim/sulfamethoxazole	+	++	[56,58,59,104]
CTLA-4	Abatacept	Trimethoprim/sulfamethoxazole, Azithromycin, Prednisone, Mycophenolate mofetil, Cyclosporin A, Rituximab, Sirolimus	+++	++	[43,45,46,105]
LRBA	Abatacept	Trimethoprim/sulfamethoxazole, Azithromycin, Prednisone, Mycophenolate mofetil, Rituximab, Cyclosporin A, Sirolimus	+++	++	[40,47,48,49,50,52,106]
IKAROS	Prednisone, Mycophenolate mofetil, Cyclosporine, Chemotherapy	Trimethoprim/sulfamethoxazole	++	++	[67,68,71,73]
IPEX	Rapamycin	Trimethoprim/sulfamethoxazole Antifungal: Itraconazole, Mycophenolate mofetil, Prednisone, Cyclosporin A	+	+++	[26,27,28]

Abbreviation: +—infrequently indicated, ++—frequently indicated and supportive evidence, +++—well established.

**Table 7:** Summary of infection spectrum of immune dysregulatory disorder.

Infections	ALPS	CTLA-4	STAT3 GOF	LRBA	APDS
Bacterial	Invasive: *S. pneumoniae*; *S. agalactiae*; *Haemophilus influenza*; *Capnocytophaga cynodegmi*. Meningitis: *N. menigitidis*	Sinopulmonary infections: *H. influenzae*; *S. pneumoniae*; *S. aureus*	Recurrent sinopulmonary infection; osteomyelitis—*Pseudomonas*, *Serratia*; staphylococcal infection; otitis media.	Recurrent sinopulmonary infection: *Staphylococcus aureus*; *S. pneumoniae*; *Streptococcus viridians*; *Acinetobacter*; *Enterococcus*; *Serratia marcescens*	Recurrent respiratory infection: *S. pneumoniae*, *Haemophilus influenzae*, *Staphylococcus aureus*, *Moraxella catarrhalis*, *Pseudomonas aeruginosa*, *Klebsiella* species
Viral		EBV infection and EBV-induced Hodgkin lymphoma	RSV; hepatitis C; herpes zoster virus; human papillomavirus; molluscum; varicella	Respiratory infection: coronavirus; adenovirus; RSV Invasive viral infection: CMV, EBV Recurrent herpes infection	EBV, CMV, HSV varicella
Fungal		Candidiasis and aspergillosis	Recurrent oral candidiasis; chronic mucocutaneous candidiasis	Candidiasis	
Atypical				Infective diarrhea: *Giardia* species; *Trichostrongylus*; *Trichomonas hominis*	
Mycobacterial		Pulmonary and esophageal tuberculosis	*M. abscessus*		

## Data Availability

Not applicable.
